# Baseline serum TSH levels predict the absence of thyroid dysfunction in cancer patients treated with immunotherapy

**DOI:** 10.1007/s40618-020-01480-6

**Published:** 2020-12-26

**Authors:** L. Brilli, R. Danielli, M. Campanile, C. Secchi, C. Ciuoli, L. Calabrò, T. Pilli, A. Cartocci, F. Pacini, A. M. Di Giacomo, M. G. Castagna

**Affiliations:** 1 Endocrinology Unit, Department of Medical, Surgical and Neurological Sciences, University Hospital of Siena, Siena, 53100 Italy; 2 Center for Immuno-Oncology, Medical Oncology and Immunotherapy, Department of Oncology, University Hospital of Siena, 53100 Siena, Italy; 3 Department of Medical Biotechnologies, University of Siena, Siena, 53100 Italy; 4 Department of Biomedical Sciences, Humanitas Clinical and Research Center, IRCCS, 20089 Rozzano, Milan Italy; 5 Department of Medical Sciences, University of Siena, Policlinico Santa Maria Alle Scotte, Viale Bracci 16, 53100 Siena, Italy

**Keywords:** Immunotherapy, Thyroid dysfunction, TSH, Anti-thyroid antibodies, PD-1/PD-L1

## Abstract

**Purpose:**

Immunotherapy against immune checkpoints has significantly improved survival both in metastatic and adjuvant setting in several types of cancers. Thyroid dysfunction is the most common endocrine adverse event reported. Patients who are at risk of developing thyroid dysfunction remain to be defined. We aimed to identify predictive factors for the development of thyroid dysfunction during immunotherapy.

**Methods:**

This is a retrospective study including a total of 68 patients who were treated with immune checkpoint inhibitors (ICIs) for metastatic or unresectable advanced cancers. The majority of patients were treated with anti-PD1 drugs in monotherapy or in combination with anti-CTLA4 inhibitors. Thyroid function and anti-thyroid antibodies, before starting immunotherapy and during treatment, were evaluated. Thyroid ultrasound was also performed in a subgroup of patients at the time of enrolment in the study.

**Results:**

Eleven out of 68 patients (16.1%) developed immune-related overt thyroid dysfunction. By ROC curve analysis, we found that a serum TSH cut-off of 1.72 mUI/l, at baseline, had a good diagnostic accuracy in identifying patients without overt thyroid dysfunction (NPV = 100%, *p* = 0.0029). At multivariate analysis, both TSH and positive anti-thyroid antibodies (ATAbs) levels, before ICIs treatment, were independently associated with the development of overt thyroid dysfunction during immunotherapy (*p* = 0.0001 and *p* = 0.009, respectively).

**Conclusions:**

Pre-treatment serum TSH and ATAbs levels may help to identify patients at high risk for primary thyroid dysfunction. Our study suggests guidance for an appropriate timely screening and for a tailored management of thyroid dysfunctions in patients treated with ICIs.

## Introduction

Immune checkpoint inhibitors (ICIs), specifically targeting the programmed cell death signaling pathway (PD-1/PD-L1), have significantly improved the outcome of several advanced cancers. Since immune checkpoints are involved in the immunological tolerance towards the self-antigens, it is not surprising that their inhibition may lead to the activation of the T cells and the development of the immune-related adverse events (irAEs) that can virtually affect every apparatus [1–3]. Randomized clinical trials reported a prevalence of endocrine irAEs during treatment with PD-1 inhibitors in monotherapy ranged from 3.8 to 20.8%, rising to 14.4–34% in case of combined treatment (anti-PD1+ anti-CTLA4) [4]. Thyroid toxicity is the most common endocrine adverse event reported [5–11]. In most cases, patients show a transient thyrotoxicosis followed by hypothyroidism, thereby resembling the course of a classical thyroiditis, but the pathogenesis is still unclear [5, 12, 13]. Currently, it is not possible to predict which patient, who is started on ICIs, is at risk to develop thyroid dysfunction. The potential relationship between the anti-thyroid antibodies levels (ATAbs), at baseline and/or during follow-up, and the development of thyroid dysfunction has been investigated in some studies and no association was found [14–19].

On the contrary, three studies reported an increased risk of thyroid dysfunction in patients with positive anti-thyroid-peroxidase antibodies (TPOAbs) [20–22] and in one study, anti-thyroglobulin antibodies (TgAbs) were significantly associated with the development of thyroid dysfunction [23]. Conversely, the clinical relevance of thyroid-stimulating hormone levels (TSH), at the baseline, has been less investigated and, in one study conducted in 73 cancer patients treated with ICIs, a significant association between TSH and the risk of developing thyroid dysfunction has been demonstrated [16]. Recently, Olsson-Brown et al. reported that patients who developed hypothyroidism without the transient thyrotoxicosis phase had TSH levels, at the baseline, significantly higher than those observed in patients who presented the typically biphasic pattern (thyrotoxicosis followed by hypothyroidism), but no comparison of TSH levels between patients with or without thyroid dysfunction was made [24]. Therefore, we aimed to assess the prevalence of primary thyroid dysfunction in a cohort of consecutive cancer patients treated with ICIs and to identify the prognostic factors able to predict the development of primary thyroid dysfunction.

## Patients and methods

### Study population

We retrospectively collected data from 68 patients who were consecutively treated ICIs at the Immuno-Oncology Center of Siena, Italy, from March 2017 to October 2019. This population was characterized by the following features: (1) absence of pre-existing thyroid dysfunction; (2) completion of at least four consecutive cycles of treatment with ICIs; (3) assessment of thyroid hormonal and ATAbs levels in the same laboratory (Endocrinology Unit, University Hospital of Siena); and (4) absence of central hypothyroidism. The median age was 58.5 years (range 27–82) and the female-to-male ratio 26/42. Thirty-nine/68 (57.3%) patients were affected by melanoma, 7/68 (10.3%) subjects by lung cancer, 6/68 (8.9%) patients by pleural mesothelioma and 16/68 (23.5%) subjects by other types of solid cancer. Most patients were treated with anti-PD1 drugs in monotherapy [45/68 patients (66.1%)], or in combination with anti-CTLA4 inhibitors [16/68 patients (23.5%)]. The remaining 7/68 (10.3%) patients were treated with anti-PDL1. A written consent was given by all patients and data were collected anonymously.

### Methods

Measurement of TSH (reference range 0.4–4 mcU/ml) and free thyroxine (FT4; reference range 5.8–16.4 pg/ml) levels was performed at baseline and during treatment.

Additionally, most patients were tested for the presence of anti-thyroglobulin (TgAbs) and anti-thyroid peroxidase antibodies (TPOAbs) before starting immunotherapy and during treatment. TPOAbs and TgAbs were classified as negative if < 35 IU/l and < 45 IU/l, respectively. Moreover, thyroid ultrasound was performed in a subgroup of patients at the time of enrolment in the study (57/72, 79.2%). TSH, FT4, TPOAbs, TgAbs, cortisol and ACTH were assayed by chemiluminescence (Immulite 2000; Diagnostic Products Corporation, Los Angeles, CA). Thyroid ultrasound was performed by the same operator with a Doppler color apparatus (AU 590 Asynchronous, EsaoteBiomedica, Florence) and a 7.5-MHz linear probe. For each patient, thyroid volume was estimated using the ellipsoid formula (width × thickness × length × 0.524). Hypoechogenicity was examined within both thyroid lobes and was revealed by comparison of thyroid parenchyma with the echo distribution of surrounding neck muscles.

### Diagnostic criteria

Thyroid dysfunction was scored as ‘subclinical’ when TSH levels were increased (subclinical hypothyroidism) or decreased (subclinical thyrotoxicosis) and FT4 values were normal. Thyroid dysfunction was, instead, scored as ‘overt’ when TSH levels were increased and FT4 values decreased (overt hypothyroidism), or vice versa (overt thyrotoxicosis). Patients were defined as having positive ATAbs if TPOAb and/or TgAb were positive.

### Definition of times to onset

Thyroid function tests were performed before drug administration, on day 1 of each cycle, repeated every 2 or 3 weeks. The time to the thyroid dysfunction onset was defined as the number of days elapsed between the first ICI administration and the date of the thyroid dysfunction detection.

### Statistical analysis

For the statistical analysis, patients were divided into two groups: the overt thyroid dysfunction group (*n* = 11) and the non-thyroid dysfunction group (*n* = 61). The *t* test for independent data or the Mann–Whitney test was performed for normal or non-normal variables, respectively. To evaluate significant differences in data frequency, we analyzed contingency tables. Tables with size larger than 2 × 2 were examined by the Chi-squared test or a numerical approximation of the Fisher exact test, when all cell frequencies were greater than 4 or not, respectively. The following variables were studied by univariate and multivariate analysis: age, sex, cancer type, ultrasonographic thyroid features, drug administered, TSH, FT4, TgAbs and TPOAbs levels at baseline and length of follow-up. A receiver operating characteristic (ROC) curve was constructed to identify a baseline TSH cut-off associated with increased risk of overt thyroid dysfunction. Statistical analysis was performed using the software StatView for Windows version 5.0.1 (SAS Institute, Cary, NC) and the IBM *SPSS* Statistics version 22.0. A *p* value < 0.05 was considered statistically significant.

## Results

### Clinical features of thyroid dysfunction induced by ICIs

**Fig. 1:**
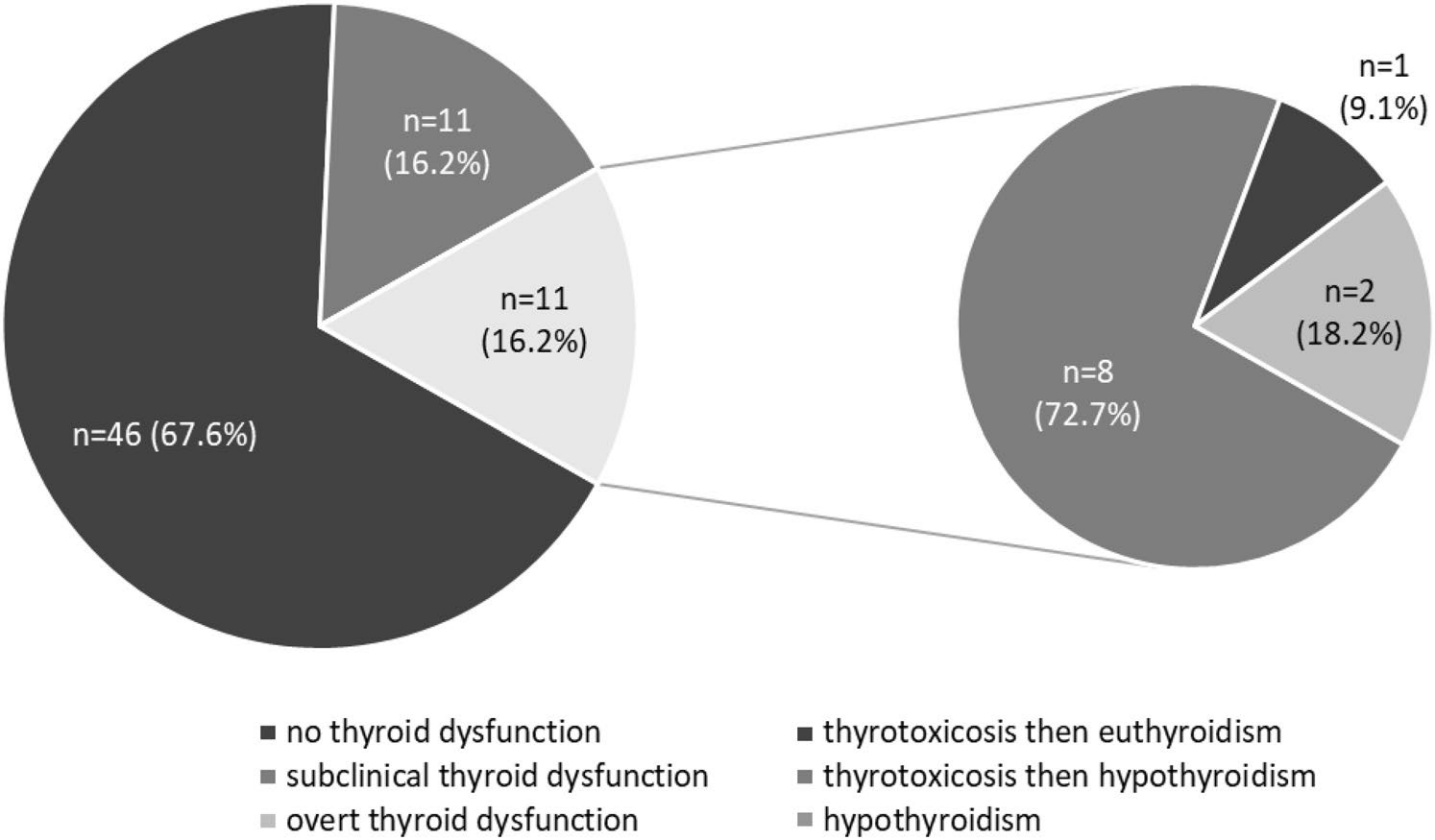
Distribution of thyroid dysfunction (*n* = 68) in the whole population and clinical presentation of thyroid dysfunction in the subgroup of patients with overt thyroid dysfunction (*n* = 11)

During the study period (median follow-up 160 days, range 49–658 days), 22/68 patients (32.3%) developed thyroid dysfunction and 11 out of them (50%) showed an overt thyroid dysfunction. A transient thyrotoxicosis was observed in 8 out of 11 patients (72.7%) with overt thyroid irAEs. These patients were all asymptomatic, not requiring any medication during the thyrotoxicosis phase, and all developed overt hypothyroidism. Moreover, we observed the occurrence of hypothyroidism, without a previous phase of thyrotoxicosis, in 2 patients (18.2%) and thyrotoxicosis, which resolved spontaneously during follow-up, in one patient (9.1%) (Fig. 1). Median time to the development of any thyroid dysfunction was 28 days (range 14–133 days), but for overt cases, the range was smaller (range 21–92) No patient required to discontinue or postpone ICIs administration due to thyroid dysfunction and all patients with overt hypothyroidism started l-thyroxine treatment.

### Association between thyroid dysfunction and thyroid hormone levels at baseline

**Fig. 2:**
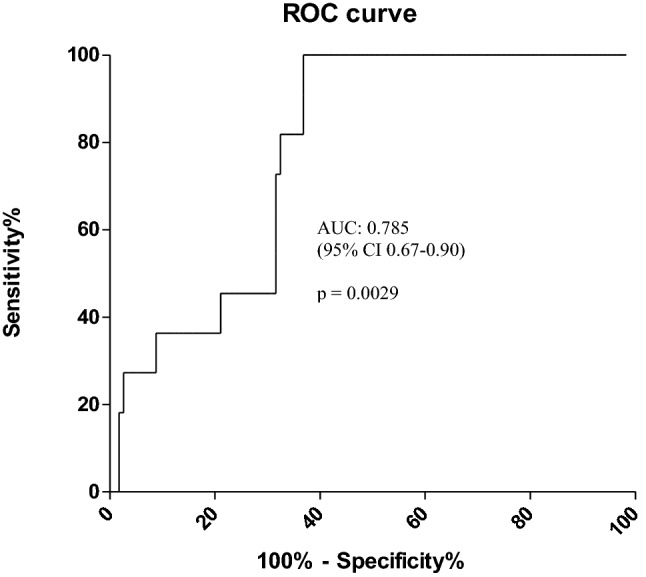
Predictive value of baseline serum TSH for overt thyroid dysfunction in cancer patients treated with ICIs by ROC curve analysis. The results indicated baseline serum TSH as a potential predictive factor for overt thyroid dysfunction with an AUC of 0.785, 95%CI of 0.67–0.90, a cut-off value of 1.72 mU/l, a sensitivity of 100% and a specificity of 63.1% respectively (*p* = 0.0029)

No significant differences were observed for FT4 levels at baseline between patients with and without overt thyroid dysfunction [median 8.2 pg/ml (range 6–11.2 pg/ml) and 9.7 pg/ml (range 6.1–13.7 pg/ml), respectively, *p* = 0.07]. In patients who developed overt thyroid dysfunction, baseline serum TSH levels were significantly higher [median 2.6 mU/l (range 1.7–3.9 mUI/l)] than those seen in patients without overt thyroid dysfunction [median 1.6 mU/l (range 0.4–3.9 mUI/l)] (*p* = 0.003) (Table 1). By ROC curve analysis, we found that a serum TSH cut-off of 1.72 mUI/l had a good diagnostic accuracy in identifying patients without overt thyroid dysfunction during follow-up [area under the ROC curve (AUC) 0.785, *p* = 0.0029] (Fig. 2). Specifically, none of the patients with TSH levels < 1.72 mUI/l developed overt thyroid dysfunction [sensitivity and negative predictive value (NPV) of 100%] while 11/32 patients (34.4%) with serum TSH levels ≥ 1.72 mUI/l had overt thyroid dysfunction during follow-up [specificity of 63.1% and positive predictive value (PPV) of 34.3%].

**Table 1:** Baseline characteristics according to the presence/absence of overt thyroid dysfunction

Characteristics	Overt Thyroid dysfunction	Non-overt thyroid dysfunction	*p* value
	*n* = 11	*n* = 57	
Age (years)			
Median	51	60	0.13
Range	29–72	27–82	
Sex			
Females	7 (63.6%)	19 (33.3%)	0.08
Male	4 (36.4%)	38 (66.6%)	
Tumor type			
Melanoma	8 (72.7%)	31 (54.4%)	0.39
Lung	1 (9.1%)	6 (10.5%)	
Mesothelioma	0 (0%)	6 (10.5%)	
Others	2 (18.2%)	14 (24.6%)	
ICIs			
PD1	9 (81.8%)	36 (63.1%)	0.44
PD1 + CTLA4	2 (18.2%)	14 (24.6%)	
PDL1	0 (0%)	7 (12.3%)	
Pre-existing TPOAbs^1^			
Positive	3 (30%)	1 (1.9%)	**0.01**
Negative	7 (70%)	52 (98.2%)	
TSH at baseline (mUI/L)			
Median	2.6	1.6	**0.003**
Range	1.7–3.9	0.40–3.9	
FT4 at baseline (pg/ml)			
Median	8.2	9.7	0.07
Range	6–11	6.1–13.7	
Thyroid volume at baseline (ml)			
Median	12	14	0.25
Range	7.9–11	6–45.7	
Thyroid hypoechogenicity^2^			
Yes	3 (27.2%)	5 (11.4%)	0.17
No	8 (72.8%)	39 (88.6%)	
Follow-up median (days)	133	164	0.43

^1^Baseline TPOAbs available in 63 patients

^2^Thyroid ultrasound performed at baseline in 55 patients

Significant *p* values are in bold

### Association between thyroid dysfunction and anti-thyroid antibodies (at baseline and during follow-up)

ATAbs were tested at baseline and during follow-up in 63/68 patients (92.6%); only 4/63 (6.3%) were positive for TPOAbs alone and nobody had baseline positive TgAbs. A significant correlation was found between ATAbs status and the development of overt thyroid dysfunction (*p* = 0.0008). Specifically, 30% of patients with overt thyroid dysfunction (3/10) had positive ATAbs at baseline, while only 1.9% of patients (1/53) without thyroid dysfunction had positive ATAbs at baseline (*p* = 0.01) (Table 1). Furthermore, we analyzed the changes of ATAbs over the time and follow-up data were available in all patients (*n* = 59) with negative ATAbs and in 3/4 patients (75%) with positive ATAbs at baseline. In the 3 ATAbs-positive patients, a marked increase of ATAb levels during follow-up was observed and all of them developed an overt thyroid dysfunction. In 89.8% of patients (53/59) with negative ATAbs, the antibodies levels did not change from baseline to the end of follow-up; while in 10.2% of patients (6/59), thyroid-specific antibodies turned positive after a median follow-up of 59.5 days (range 43–140 days). Four/six patients with positive ATAbs during follow-up (66.6%) developed an overt thyroid dysfunction. Thyroid dysfunction occurred before the appearance of ATAb positivity (24 days versus 56 days from the beginning of ICIs).

Out of 53 patients with persistent negative ATAbs during follow-up, 4 patients (7.5%) developed an overt thyroid dysfunction; while in the remaining 49 patients (92.5%), no thyroid dysfunction was documented during follow-up. In all of them, persistent negative ATAbs were observed at last of follow-up, with a median time-lapse from diagnosis of thyroid dysfunction to last follow-up of 160 days.

**Fig. 3:**
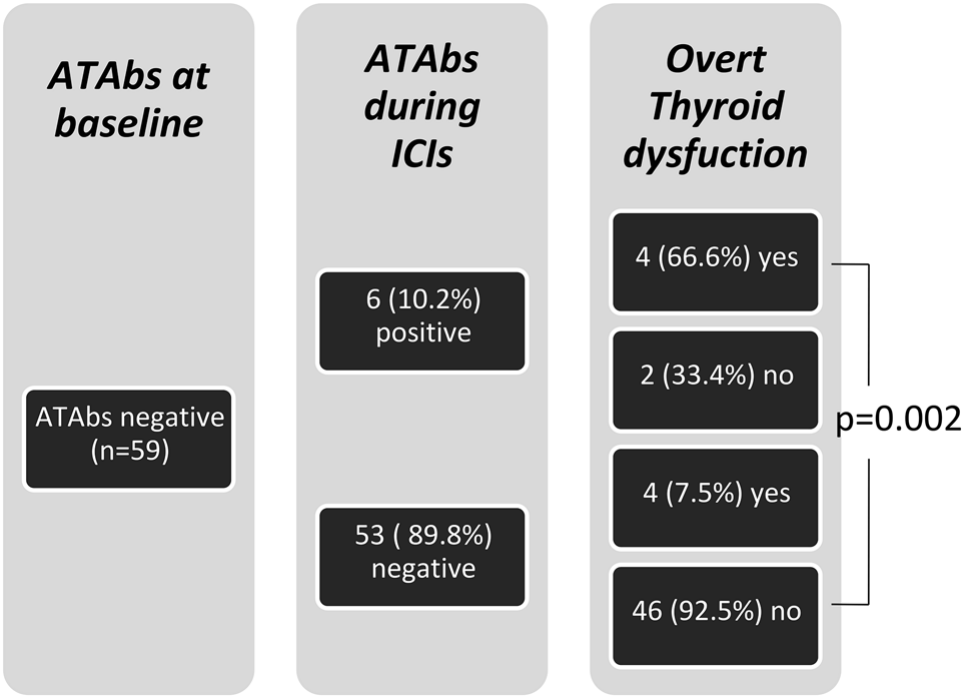
Anti-thyroid antibodies (ATAbs) during follow-up in 59 patients with negative ATAbs at baseline

A significant higher rate of overt thyroid dysfunction during follow-up was observed in the subgroup of patients who developed positive ATAbs (66.6%) when compared with the subgroup of patients with negative ATAbs (7.5%, *p* = 0.002) (Fig. 3).

### Association between thyroid dysfunction and thyroid ultrasonography features at baseline

Thyroid ultrasound was performed in 55/68 patients (80.9%) at baseline. An ultrasound pattern with diffuse hypoechogenicity was present in 8 patients (14.5%), while thyroid nodules greater than 1 cm were observed in 11 patients (20.0%). The median thyroid volume was 13 ml (6–45.7 ml). Thyroid ultrasound data were available in all patients who developed an overt thyroid dysfunction (n = 11) and in 44/57 (77.1%) patients without thyroid dysfunction during follow-up. No differences in the hypoechogenicity (*p* = 0.17), presence of thyroid nodules (*p* = 1.0) and thyroid volume (*p* = 0.25) were observed between patients with and without overt thyroid dysfunction (Table 1).

### Risk factors for the development of overt thyroid dysfunction during ICIs therapy (univariate and multivariate analysis).

There was no difference in age (*p* = 0.13), sex (*p* = 0.08), cancer type (*p* = 0.39), ultrasonographic thyroid features (*p* = 0.17 for echogenicity and 0.25 for thyroid volume), type of ICIs (*p* = 0.44) and length of follow-up (p = 0.43) between patients who developed thyroid dysfunction and those who did not, at univariate and multivariate analysis. On the contrary, TSH and positive ATAb levels, at baseline, were significantly associated with the development of overt thyroid dysfunction during follow-up at univariate (*p* = 0.003 and *p* = 0.01, respectively) and at multivariate analysis (*p* = 0.0001 and *p* = 0.009, respectively).

## Discussion

Thyroid dysfunction is common during ICIs treatment, with hypothyroidism and thyrotoxicosis being the most frequent endocrinological manifestations (5–11). In our study, any type of thyroid dysfunction was observed in 32.4% of cancer patients, while an overt dysfunction in 16.2% of the cohort. Our results agree with most published studies [15, 17, 18, 25, 26]. Specifically, in a retrospective monocentric study including a large number of patients, the rate of all thyroid dysfunctions was of 30% with the lowest rate found in patients treated with ipilimumab in monotherapy (23%), an intermediate rate in those treated with anti-PD-1 in monotherapy (39%) and the highest in those treated with a combination therapy (ipilimumab + nivolumab) (50%) [27]. Similar findings were observed in another large prospective study that reported a higher rate of thyroid irAEs during combined treatment compared to monotherapy (22% versus 9%) [25]. Conversely, we did not find a significant correlation between the different therapeutic approaches. In particular, the prevalence of overall (overt and subclinical) thyroid dysfunction was 33.3% in patients treated with anti-PD1 inhibitors in monotherapy and 25% in those on combined treatment (ipilimumab + nivolumab); the latter result is not consistent with the previous studies likely due to the small number of patients treated with combined therapy in our series (only 16 patients, 22.2% of the whole cohort). However, a recent retrospective study, conducted in a cohort of 1146 individuals without pre-existing thyroid disease, does not highlight any association between thyroid irAEs and specific ICIs [28].

Currently, it is not possible to identify patients at risk of thyroid dysfunction during treatment with ICIs. The role of thyroid antibodies in the pathogenesis and/or in predicting an increased risk of ICIs-induced thyroid abnormalities remains to be defined. Some studies did not observe any association [14–19], while recent studies have reported an increased risk of thyroid dysfunction in patients with positive TPO antibodies [20–22] or TgAbs [23]. In our cohort of patients, we found a significantly higher rate of positive TPOAbs, at the baseline, in patients with thyroid dysfunction (30%) compared to patients without thyroid dysfunction (1.9%). However, our data suggest that thyroid antibodies are infrequently associated with immune checkpoint-induced thyroid dysfunction, since most patients with thyroid dysfunction had negative ATAbs (70.0%, *p* = 0.01). On the other hand, the positivity of TPOAbs at baseline predicts an increased risk of thyroid dysfunction following treatment with ICIs (75% of overt thyroid dysfunction in TPOAbs positive patients); therefore, a close follow-up of these patients may be required. Similarly, the conversion of ATAbs from negative to positive during the follow-up was associated with a higher rate of overt thyroid dysfunction (66.6% versus 7.5% in patients with negative thyroid antibodies at baseline and during ICIs therapy), but, interestingly, the switch occurred after the onset of the thyroid dysfunction. This time lapse suggests that the appearance of anti-thyroid antibodies could be due to the humoral response toward the release of thyroid antigens during a destructive thyroiditis and, therefore, representing an epiphenomenon. This hypothesis may be, also supported, by the observation that a thyrotoxicosis phase was documented before most cases who developed an overt thyroid dysfunction (72.7%). Based on these data, we may speculate that the occurrence of hypothyroidism, rather than an immune-mediated event, is a result of a massive destruction of the thyroid due to a toxic effect of ICI. However, the thyroid antibodies positivity before treatment confers susceptibility for the hypothyroidism development as we observed in our study. Also, serum TSH levels, at the baseline, were significantly associated with the development of overt thyroid dysfunction under ICIs treatment. We demonstrated that patients with TSH < 1.7 mU/l (sensitivity and NPV of 100%), at the baseline, did not develop overt thyroid dysfunction suggesting that in case of TSH below this cut-off, additional tests during ICIs therapy may be not routinely performed at each cycle. Recently, Pollack et al. reported that a TSH > 2.19 mU/l, at the baseline, was significantly associated with the development of thyroid dysfunction [17]. However, applying this cut-off for predicting the occurrence of thyroid dysfunction may affect its utility in the clinical practice because of the high rate of false positives (about 30%) and negatives (about 50%) results. On the contrary, our TSH cut-off has a very high NPV value with no false-negative results. Therefore, using this TSH cut-off, we may be able to avoid unnecessary tests in the subgroup of patients with a baseline serum TSH < 1.72 mU/l, without missing cases of overt thyroid dysfunction during ICIs therapy.

Some limitations of this study are intrinsic to its retrospective design, the presence of multiple types of cancer, the use of different ICIs and the small sample size. However, the data have several strengths: a standardized clinical and biochemical assessments in the same institution, thyroid dysfunction was well characterized and all patients were systematically followed up, data regarding thyroid antibodies were available in the majority of patients at the baseline and during the follow-up, allowing a better understanding of the relationship between autoimmunity and thyroid dysfunction observed during ICIs therapy and finally, to our knowledge this is the first study that evaluated the trend of ATAbs in a significant proportion of cancer patients treated with ICIs.

## Conclusion

We recommend assessing TSH and free T4 levels, before starting ICIs therapy, in accordance with all published guidelines [29–31]. In patients with TSH < 1.72 mU/l, additional test may be avoided unless signs and/or symptoms of thyroid dysfunction appear during follow-up. On the contrary, in patients with basal TSH > 1.72 mU/l, serum ATAbs should be assessed, before the first cycle of ICI treatment, given the higher risk of overt thyroid dysfunction in the presence of positive thyroid antibodies (about 75%). Finally, in patients with serum TSH > 1.72 mU/l and negative TPOAb, the risk for developing thyroid dysfunction is lower (about 30% of cases, data not showed); therefore, in these cases, a less intensive follow-up may be adequate.

Our study suggests guidance for an appropriate timely screening and for a tailored management of thyroid dysfunctions in patients treated with immune checkpoint inhibitors. Additional prospective studies with a larger cohort of patients are needed to confirm our results.

## Data Availability

The datasets generated during and/or analyzed during the current study are available from the corresponding author on reasonable request.
